# ROS mediated anticandidal efficacy of 3-Bromopyruvate prevents vulvovaginal candidiasis in mice model

**DOI:** 10.1371/journal.pone.0295922

**Published:** 2023-12-28

**Authors:** Ravi Jothi, Seong-Tshool Hong, Munkhtur Enkhtsatsral, Shunmugiah Karutha Pandian, Shanmugaraj Gowrishankar

**Affiliations:** 1 Department of Biotechnology, Science Campus, Alagappa University, Karaikudi, Tamil Nadu, India; 2 Department of Biomedical Sciences and Institute for Medical Science, Jeonbuk National University Medical School, Jeonju, South Korea; Universidad San Francisco de Quito, ECUADOR

## Abstract

Candidal infections, particularly vulvovaginal candidiasis (VVC), necessitate effective therapeutic interventions in clinical settings owing to their intricate clinical nature and elusive understanding of their etiological mechanisms. Given the challenges in developing effective antifungal therapies, the strategy of repurposing existing pharmaceuticals has emerged as a promising approach to combat drug-resistant fungi. In this regard, the current study investigates molecular insights on the anti-candidal efficacy of a well-proven anticancer small molecule -3-bromopyruvate (3BP) against three clinically significant VVC causing *Candida* species *viz*., *C*. *albicans*, *C*. *tropicalis* and *C*. *glabrata*. Furthermore, the study validates 3BP’s therapeutic application by developing it as a vaginal cream for the treatment of VVC. 3BP exhibited phenomenal antifungal efficacy (killing >99%) with minimum inhibitory concentrations (MIC) and minimum fungicidal concentrations (MFC) of 256 μg/mL against all tested *Candida* spp. Time killing kinetics experiment revealed 20 min as the minimum time required for 3BP at 2XMIC to achieve complete-killing (99.9%) in all *Candida* strains. Moreover, the ergosterol or sorbitol experiment explicated that the antifungal activity of 3BP does not stem from targeting the cell wall or the membrane component ergosterol. Instead, 3BP was observed to instigate a sequence of pre-apoptotic cascade events, such as phosphatidylserine (PS) externalization, nuclear condensation and ROS accumulations, as evidenced by PI, DAPI and DCFH-DA staining methods. Furthermore, 3BP demonstrated a remarkable efficacy in eradicating mature biofilms of *Candida* spp., achieving a maximum eradication level of 90%. Toxicity/safety profiling in both *in vitro* erythrocyte lysis and *in vivo Galleria mellonella* survival assay authenticated the non-toxic nature of 3BP up to 512 μg/mL. Finally, a vaginal cream formulated with 3BP was found to be effective in VVC-induced female mice model, as it significantly decreasing fungal load and protecting vaginal mucosa. Concomitantly, the present study serves as a clear demonstration of antifungal mechanistic action of anticancer drug -3BP, against *Candida* species. This finding holds significant potential for mitigating candidal infections, particularly VVC, within healthcare environments.

## Introduction

Vulvovaginal candidiasis (VVC) is a universal distressing disease that primarily affects women of reproductive age [1]. *Candida albicans* is the primary cause of VVC, while non-albicans species such as *Candida krusei*, *Candida tropicalis* and *Candida glabrata* can occasionally contribute [2, 3]. Despite the fact that RVVC is not associated with mortality, the financial burden and morbidity related to medical care have been rapidly rising each year [4]. Nevertheless, till to date, the risk factors associated with increased susceptibility to RVVC have not been clearly elucidated. The involvement of drug resistant strains in the pathogenesis is the major challenge faced during the RVVC treatment [5]. This startling clinical situation is being attributed to the risk factors associated with modern medical practices, including the usage of broad-spectrum antibiotics, immunosuppressive drugs and medical implants. These practices pose a serious threat to the health of women, especially in those who have HIV or immune systems that are compromised.

Despite the high prevalence of candidal infections, current treatment options are limited to three antifungal drug classes: azoles, polyenes, and echinocandins [6, 7]. Triazoles have been widely used in the treatment of clinical *Candida* infections because of their superior safety profile, oral absorption and broad-spectrum antifungal activity [8]. Concomitant with prolonged usage of drug and the pathogen’s ability to proliferate into biofilm on various biotic and abiotic surfaces, there has been increasing emergence of drug resistance to these conventional antifungals [9, 10]. Since growing biofilm cells are 1000 times more resistant than planktonic cells, biofilm formation by *C*. *albicans* acts as a defender to protect it from the action of host immunity and antimicrobial agents [11].

The increased prevalence of drug resistance to available antifungal agents in VVC is undoubtedly caused for concern over the development of new antifungal candidates [12]. However, drug development through de novo drug discovery requires many clinical trials to ensure its usage in medical settings; this is likely to be time- and money-consuming, and it limits novel drugs from being employed fast in clinical settings [13, 14]. A multitude of proof-of-concept studies have been conducted in support of alternative drug repurposing strategies to envisage antibacterial properties of drugs approved for use in a variety of therapies, including anticancer, antifungal and cardiovascular therapy [15, 16].

3-bromopyruvate (3BP) -a halogenated derivative of pyruvic acid known for its strong alkylating properties, has long been one of the most well researched compounds in the category of halogenated carboxylic acids. In the past decade, 3BP has been well documented/established for its exceptional cytotoxic effect on cancer cells, including hepatocarcinomas, colon, breast and lung malignancies [17, 18]. The selective and cancer cell specific toxicity of 3BP has been attributed as an imperative anticancer candidate, and is still used as a last-resort treatment in a few cancer clinics throughout the world [19]. Because 3BP has no negative effects on healthy cells [20], it is tempting to consider as a potent alternative antimicrobial agent for the treatment of invasive infections caused by antibiotic resistant pathogens. Given the significance of 3BP’s non-toxicity and lack of detrimental effects on healthy human cells, it is intriguing to examine 3BP as a potent alternative antimicrobial agent for the treatment of VVC caused pathogens that are resistant to common antimicrobials.

More importantly, a patent application (WO2011127200A8) has been filed for the use of 3BP’s anti-glycolytic action in the treatment of bacterial infections [21]. Considering the 3BP’s established pharmacological and toxicological characteristics, the current research study thoroughly examines the antifungal mechanistic action of a well-known anticancer drug, 3BP, against three clinically significant VVC causing *Candida* species, namely *C*. *albicans*, *C*. *tropicalis*, and *C*. *glabrata*. Besides, attempts were also undertaken to evaluate the *in vitro* and *in vivo* anti-infective efficacies of 3BP-formulated vaginal cream for the treatment of VVC using female mice model.

## Materials and methods

### Strains and culture conditions

Three strains of *Candida*—*C*. *albicans* (ATCC 10231), *C*. *glabrata* (MTCC 184), and *C*. *tropicalis* (MTCC 3019)—that were purchased from HiMedia in India were utilized in the investigation. Before being employed in experiments, the strains were routinely grown at 37°C in yeast extract peptone dextrose (YEPD) broth and kept in sabouraud dextrose agar (SDA) plates. The 3 h culture with 0.1 optical density (OD) (1 × 10^6^ CFU/mL) was used as inoculum to perform all *in vitro* assays [11].

### Compound preparation

The compound 3BP was obtained from Sigma-Aldrich. The stock solution was prepared at a final concentration of 100 mg/mL using ethanol as a solvent. Finally, the stock was stored at 4°C until further use.

### Determination of minimal inhibitory concentrations

The MIC of 3BP against *C*. *albicans*, *C*. *glabrata* and *C*. *tropicalis* was determined using a broth microdilution assay as demonstrated by Gowrishankar *et al*., [22]. In brief, 1 mL of YEPD medium (HiMedia, India) was dispensed into a 24-well Microtitre Plate (MTP). The 3BP solution (2048 μg/mL) was then added to the first well, which contained 2 mL of YEPD broth. One mL of medium was serially diluted in the subsequent wells to achieve a 3BP concentration ranging from 0 to 1024 μg/mL. Finally, 1% of exponential cultures corresponding to each fungal strain were added in different wells, and the plate was stored at 37°C for 24 h. The wells containing YEPD medium with and without culture (devoid of the test compound) were served as control and blank, respectively. Subsequent to incubation, MIC was determined by measuring the OD at 600 nm using a spectrophotometer (Spectra Max 3, Molecular Devices, The United States). The MIC was defined as the minimal concentration of 3BP that showed visible growth inhibition in YEPD broth compared to untreated control. All the experiments were carried out in triplicate.

### Determination of minimal fungicidal concentrations

To determine the MFC, spread plate method was performed as stated by Hafidi et al., [23]. A 100 μL of aliquots from the wells that displayed observable growth inhibition from broth microdilution assay was used to spread on the YEPD agar plates, and incubated at 37°C for 48 h. After incubation, CFU was calculated. MFC was defined as the lowest concentration of 3BP that showed either no growth or less than 30 fungal colonies to obtain approximately 99–99.5% killing activity.

### Time killing kinetics

The time killing rate of 3BP against test fungal strains was evaluated as described previously by Öz et al., [24]. Initially, 1 × 10^6^ CFU/mL of *Candida* cultures were added separately in a boiling tube containing 5 mL of YEPD medium and treated with 3BP at MIC and 2XMIC. The YEPD medium containing fungal culture without 3BP served as control. The tubes were then incubated at 37°C. 200 μL and 100 μL of cell suspension were removed at the predetermined time intervals (at 30, 60, 120, 180, 210, 240, 270, 300, 330 and 360 min) and used for measuring optical density and CFU, respectively. In addition, 2 μL aliquots were used to spot on YEPD agar plates. Subsequently, the agar plates were incubated at 37°C for 48 h for CFU counting.

### Sorbitol assay

Since sorbitol has been considered as one of the fungal cell wall osmotic protection agents, the action of 3BP on candidal cell wall was assessed using sorbitol assay. The MIC assay was carried out using medium with and without sorbitol (control) to assess the possible underlying mechanism of 3BP on *Candida* cell wall [25]. Briefly, 1% of the test fungal strains were used to inculcate 1 mL of YEPD medium in wells of MTPs supplemented with various concentrations of 3BP (0–1024 μg/mL) along with 0.8 M sorbitol. The YEPD with fungal culture devoid of sorbitol was considered as a negative control. The plates were incubated at 37°C, and the difference in the MIC was assessed by measuring growth OD after 48 h.

### Ergosterol assay

To assess the potential mode of action of 3BP on fungal cell membrane -sterol (ergosterol), ergosterol assay was employed [26]. The MIC assay was performed in the presence and absence of exogenous ergosterol. In Brief, the test compounds 3BP (0–1024 μg/mL) were added into 1mL of YEPD medium containing exogenous ergosterol at a final concentration of 400 μg/mL. Then, 1% of fungal inoculum was added into each well and incubated at 37°C for 24 h. After incubation, the change in the MIC value of 3BP was evaluated by measuring the growth OD at 600nm.

### ROS estimation

The endogenous level of ROS accumulation in *Candida* species during 3BP manifestation was assessed through 2′, 7′-dichlorofluorescein diacetate (DCFH-DA) staining method [27]. The fungal cell suspension was initially adjusted to 1 × 10^6^ CFU/mL before treatment with ½ MIC and MIC of 3BP for 30 min. Subsequent to treatment, the cell suspension was stained with DCFH-DA (2 μg/mL) for 30 min at dark. Further, the cells were collected through centrifugation and dissolved in PBS. The fluorescence intensities were visualized under fluorescence microscope (Nikon Eclipse Ts2R, Tokyo, Japan) at the excitation and emission wavelengths of 488 and 540 nm, respectively.

### Cell membrane permeability assay

To investigate 3BP’s exposure mediated cell membrane damage, if any; a microscopic examination using PI stain was employed to comprehend cell membrane integrity in *Candida* species [28]. *Candida* cells were treated with 3BP at ½ MIC and MIC. After incubation for 30 min at 37°C, cells were harvested by centrifugation and suspended in PBS. Then, stained with PI (1 μg/mL) for 30 min and visualized under fluorescence microscopy (Nikon Eclipse Ts2R, Tokyo, Japan).

### DAPI staining

A DNA-specific fluorescent dye 4′-6-diamidino-2-phenylindole (DAPI) was employed to examine the nuclear fragmentation upon 3BP treatment [29]. After 30 min of treatment with 3BP at ½ MIC and MIC, the *Candida* cells were stained with DAPI (1 g/mL) for 30 min in the dark. Subsequent to incubation, the cell pellet was collected through centrifugation, and washed with PBS. The alteration of fluorescence intensity was observed under fluorescence microscopy (Nikon Eclipse Ts2R, Tokyo, Japan).

### Effect of 3BP on preformed biofilm

The eradication efficacy of 3BP on mature biofilm of *Candida* spp. was evaluated by crystal violet (CV; HiMedia, India) staining method [30]. In brief, biofilm formation was induced by culturing overnight *Candida* stains in 1 mL of spider medium supplemented with 10% of hyphal inducing medium (FBS) in 24-well MTP. After incubation, 37°C for 48 h, formed biofilm cells were treated with 3BP at various concentrations (½MIC, MIC & 2XMIC) and the plates were again incubated at 37°C for 24 h. Subsequent to incubation; the formed sessile cells on the bottom of MTP were stained with 0.4% CV for 15 min. After destaining with 15% glacial acetic acid for 15 min, the amount of CV bound biofilm cells was quantified spectrophotometrically at 570 nm. The percentage of biofilm inhibition was determined using the following formula.

The relative biofilm inhibition: % of biofilm inhibition = [(Control OD570 nm −Treated OD570 nm)/ Control OD570 nm] × 100.

### Erythrocyte lysis assay

The cytotoxicity of 3BP on human erythrocyte cells was analyzed using the protocol described previously by Turecka et al., [31]. Briefly, 5 mL of human blood was collected from healthy volunteers and transferred to a fresh 15 mL falcon tube containing an anticoagulant agent (sodium citrate). The total erythrocyte cells were harvested by centrifugation at 3000 rpm for 5 min and dissolved in PBS at the final concentration of 2%. Further, the blood suspension was exposed to different concentrations (0–1024μg/mL) of 3BP at 37°C for 1 h. Subsequent to incubation, the supernatant was collected to spectroscopically measure the erythrolytic activity at 415 nm.

### *In vivo* toxicity assessment using *Galleria mellonella* larvae

The *in vivo* toxic effect of 3BP was analyzed using the invertebrate animal model *Galleria mellonella* [32]. Larvae weighing around 0.2–0.4 g were taken for experiments. Larvae were separated randomly as following five experimental groups (5 larvae/group) Group I: Naive control, Group II: Positive control (2% methanol), Group III: vehicle control (PBS), Group IV: 3BP (MIC) and, Group V: 3BP (2XMIC). Injection was performed with a U-100 insulin syringe (Dispovan, HMD, India) in the last proleg. The larva in the vehicle control group was injected with maximum volume of solvent used to dissolve 2XMIC of 3BP. Uninjected larvae serve as the naive control group. Larva groups were incubated at 37°C for 5 days. Survival was monitored every 24 h.

### Formulation of vaginal cream containing 3BP

To prepare the vaginal cream, oil/water emulsion method was carried out. The oil phase comprising cetyl alcohol, stearyl alcohol, isopropyl myristate and polysorbate 80 was heated at 75°C until complete melting of the constituents [33]. Then, the aqueous phase consisting propylene glycol in water was mixed with oil phase at the ratio of 3:1, and stirred constantly until the temperature reaches <40°C. Then, 3BP was incorporated into the above mentioned suspension at various concentrations i.e., 0.25, 0.5 and 1% w/w. Finally, the pH of the vaginal cream was adjusted to skin pH i.e., 4.0–4.5 using lactic acid. In order to have comparative analysis with a positive/reference antifungal drug, miconazole (at 2% concentration) (HiMedia, India) based vaginal cream formulation was also developed.

### Physicochemical evaluation of vaginal cream

- **Temperature stability test**
  The optimized cream formulation was stored at both 4°C (Refrigerator) and 37°C (room temperature) to evaluate its stability under different temperatures.
- **pH test**
  The change in the pH of formulated vaginal cream was analyzed over a one month period. A total of 1 g of vaginal cream was dissolved in 10 mL of distilled water and pH was measured using a pH meter.
- **Spreadability test**
  Spreadability test was carried out by using two upper glass lids of petri dishes. One g of cream formulation was placed at the corner between two upper glass lids and 30 g weight was placed on the upper glass lid for 5 min to compress and obtain a uniform thickness of cream. The length of cream after spreading was taken as a measure of spreadability [34].

### *In vitro* antifungal efficacy evaluation

In order to assess the *in vitro* antifungal efficacy of formulated vaginal cream, agar well diffusion assay was performed [35]. Briefly, *Candida* cell suspension (1× 10^6^ CFU/mL) was swabbed uniformLy on the YEPD agar plate. Then, 0.1 g of formulated vaginal cream was aseptically placed inside the well using sterile spatula. After incubation at 37°C for 24 h, antifungal activity of cream was confirmed through a zone of inhibition around the loaded wells. This assay was carried out at the predetermined time intervals (0, 1, 7, 14, 21 and 28 days.

### Preclinical evaluation of 3BP vaginal cream in a vulvovaginal candidiasis (VVC) animal model

#### Animal selection and maintenance

Female mice (n = 25) at twelve weeks of age with a body weighing approximately 20–25 g were obtained from breeding colonies and acclimatized for 3 weeks before being used in the experiments. Mice were housed at 8 animals per cage, with free access to food (6% fat, 18% protein; Rodent RQ 18–6; Zeigler Bros., Inc.) and filtered water. They were maintained under a 12 h light/12 h dark cycle at a temperature of 24° C and humidity of 55±5%.

#### Ethical statements and humane endpoints

The animal protocols were reviewed and approved by the Ethics Committee of the Institution’s Laboratory Animal Center in compliance with the guidelines of the Ethics Committee of Jeonbuk National University Laboratory Animal Center Guidelines (Permit Number: CBU 2012–0040). A protocol for early/humane endpoints for animals was implemented according to the regulations for animal facilities regarding the identification and evaluation of pain, distress, and suffering as well as the requirement that animals that are close to death should be put to death right away. No animals died or showed signs of discomfort, distress, or suffering before the experiment was over. At the end of the experiment, the animals were killed using cervical dislocation while being sedated with ketamine and xylazine (120 mg + 10 mg per kg of body mass given intraperitoneally).

#### Animal model of VVC

To establish the infections in mice, *C*. *albicans* ATCC1231 was used [36]. The animals (n = 25; 5 per group) was divided randomly into five groups *viz*., Group I: Naive control (without any treatment); Group II: VVC (Infected group); Group III: Negative control (3BP formulation alone); Group IV: Positive control 1 (VVC+ formulation containing miconazole) and; Group V: Treatment group (VVC+ formulation containing 1% 3BP). Briefly, 0.1 mL of *C*. *albicans* culture suspension of 1 x 10^7^ CFU/mL was injected intra-vaginally into the mice using a micropipette with disposable tips. After two days of infection, the vaginal lavage was collected and diluted in a 0.85% saline, and plated on YEPD for CFU counting. The infection was considered sufficient if the mean count for the vaginal lavage cultures from each mouse was at least 10^4^ CFU/mL. The infected mice were administered intra-vaginally with in-house formulated creams (0.25 g) once in a day for 6 successive days. On 24, 48 and 72 h after treatment establishment, vaginal fungal load, especially *C*. *albicans* was evaluated by determining the CFU/mL.

#### Histopathology study

Histopathological investigations were carried out to assess the effectiveness of 3BP cream formulations in protecting against vaginal damage caused by VVC. At the end of experiment, animals were slaughtered for their vagina and processed for histopathology. The sliced vaginal sections were further stained with hematoxylin. Vaginal damage if any was examined via light microscopy.

### Statistical analysis

All the experiments were carried out in biological triplicates with at least two experimental replicates and the data were presented as mean ± standard deviation. To evaluate statistical differences between control and treated samples one-way analysis of variance (ANOVA) and Dunnett’s post hoc test was performed using SPSS statistical software 17.0. The significance was represented as *p* ≤0.05 and < 0.01, respectively.

## Results & discussion

Vaginal infections are one such long-lasting health annoyance that affects millions of women around the world [37]. Globally, the improper usage of antifungals and the drastic increase in the immunocompromised population have resulted in an unfortunate increase in the incidence of VVC [38]. Alarmingly, the emergence of antifungal resistance among *Candida* species imposes threatening challenges in the control of VVC, necessitating an urgent need for the development of new antifungal candidates with a broad-spectrum pharmacological profile. In this context, researchers working in the field of antimicrobial resistance have identified drug repurposing as a promising strategy for effectively treating infections caused by MDR pathogens [39].

On this background, the present study was focused on investigating the antifungal mechanistic action of a well-proven anticancer drug, 3BP against three clinically important *Candida* species associated with VVC. To ensure 3BP’s therapeutic applicability, a vaginal cream containing 3BP was formulated, and efforts were made to investigate its physicochemical properties as well as *in vivo* efficacy in the treatment of VVC using a female mice model.

### 3BP displays fungicidal action against planktonic cells of *Candida* species

**Fig 1:**
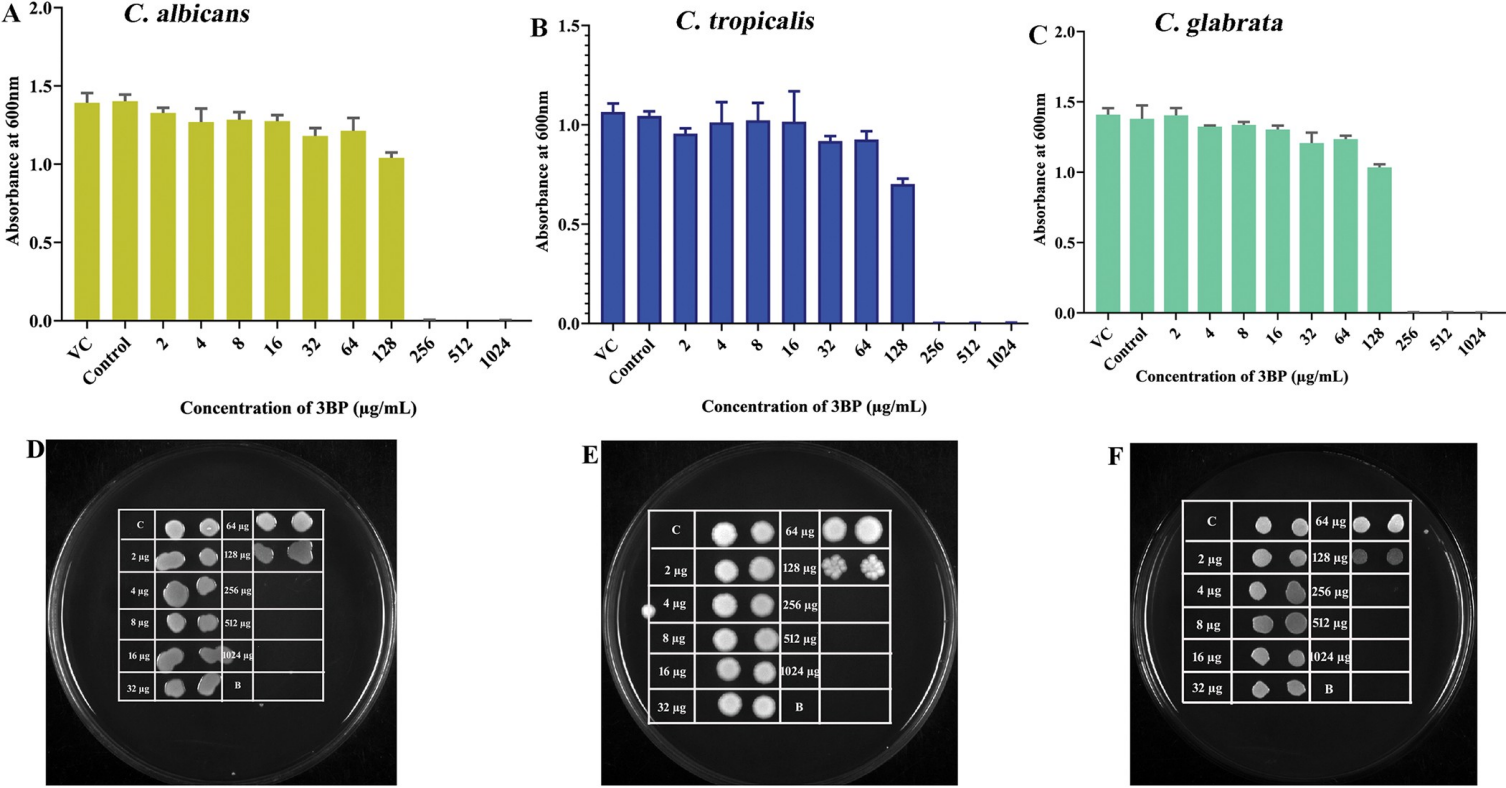
Determination of MIC of 3BP against *C*. *albicans*, *C*. *tropicalis* and *C*. *glabrata* through (a-c) spectrophotometrical and (d-f) spot method. After 24 hours of exposure to 3BP at concentrations ranging from 0–1024 g/mL, the growth OD600 was measured. VC denotes for vehicle control (ethanol at 10 μL).

The inhibitory efficacy of 3BP on the planktonic growth of three tested *Candida* species was initially evaluated by determining the MIC and MFC using broth microdilution and spread plate methods, respectively. The observed *Candida* species growth inhibition during 3BP manifestation is represented in Fig 1. The obtained MIC of 3BP was 256 μg/mL for all tested *Candida* species, indicating that 3BP’s antifungal efficacy was not species-dependent. As shown by the current results, 3BP has the potency to combat three different *Candida* species, confirming its broad-spectrum anti-*Candida* efficacy.

**Fig 2:**
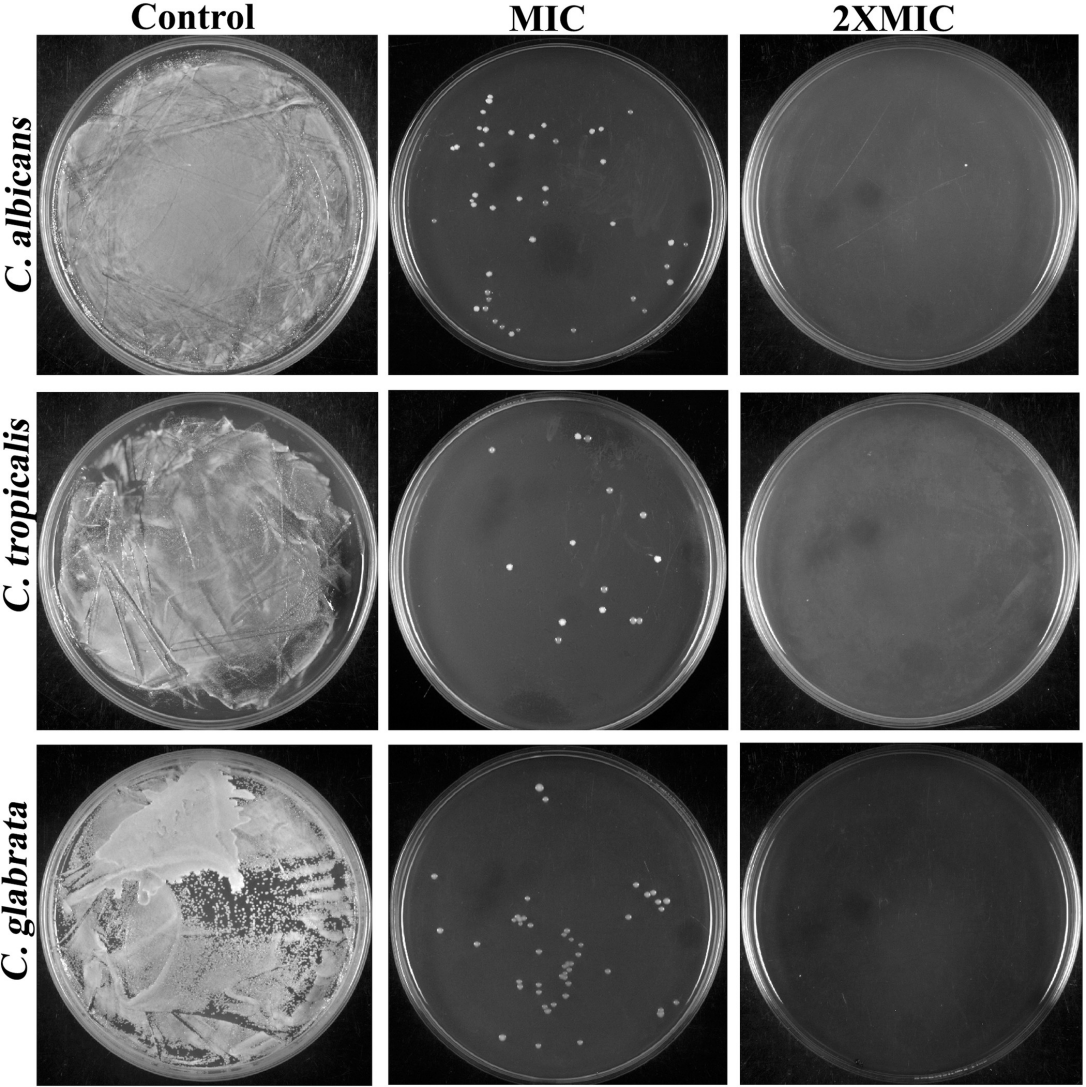
Determination of 3BP’MFC on *C*. *albicans*, *C*. *tropicalis* and *C*. *glabrata* using spread plate method.

To identify the anti-candidal nature of 3BP, either fungicidal (MFC = MIC) or fungistatic (MFC>MIC), the ratio r = MFC/MIC was utilized [24], in accordance with the limits set by the most recent National Committee on Clinical Laboratory Standards (NCCLS). As shown in Fig 2, cells exposed to the MIC of 3BP displayed less than 30 colonies on agar plates, demonstrating that there was no difference in the concentration of 3BP used to achieve the MIC and MFC. This substantiated that 3BP killed *Candida* species, rather than simply arresting their proliferation, thus demonstrating the fungicidal nature of 3BP. Thus, it is envisaged that 3BP could be an efficient antifungal drug as it invariably exhibits phenomenal broad-spectrum fungicidal effects against three clinically significant species of *Candida*.

**Fig 3:**
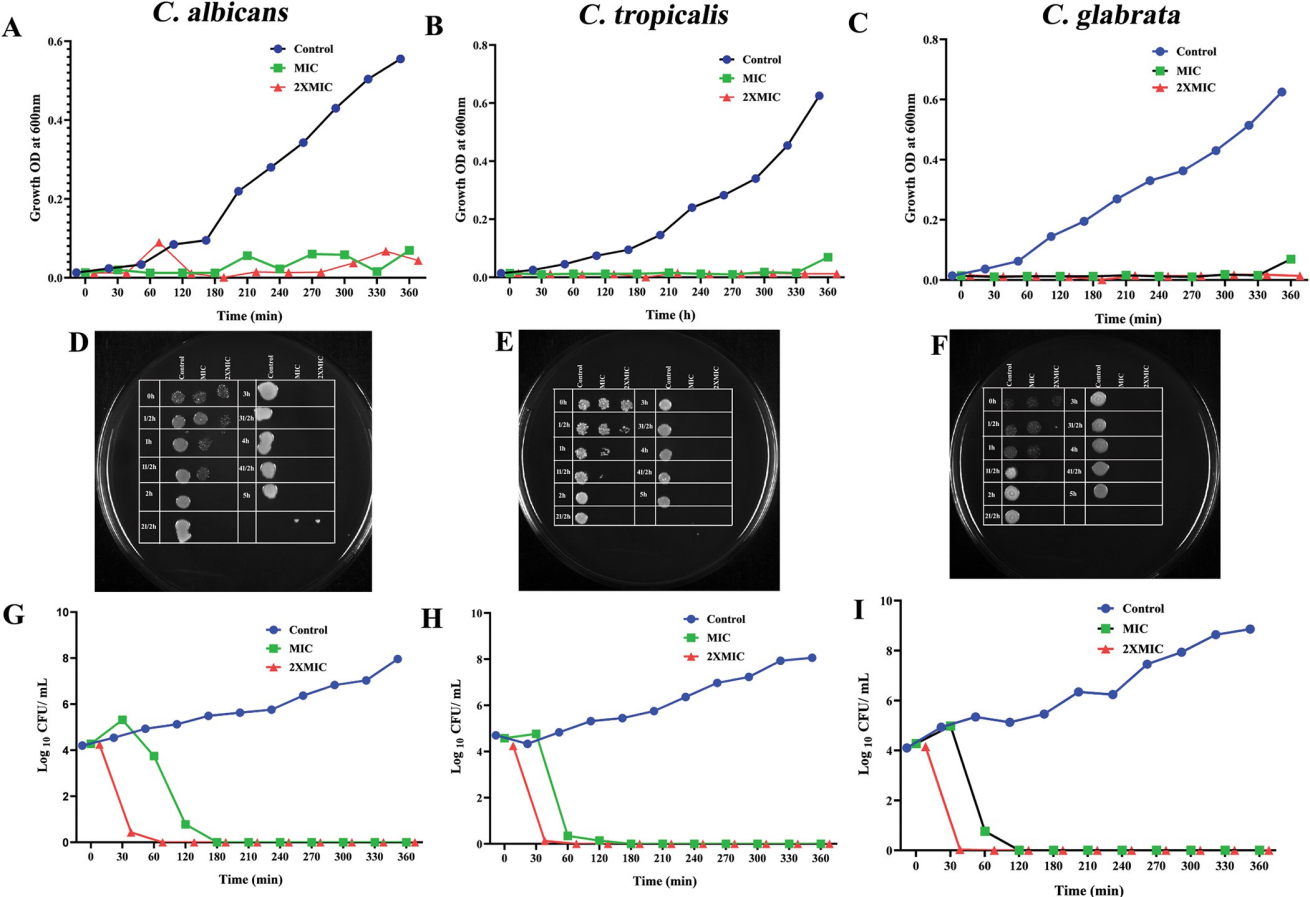
Time-kill kinetics displayed the inhibition of planktonic growth of all *Candida* strains treated with different concentration of 3BP (at MIC& 2XMIC) at various time intervals (30, 60, 90, 120,180, 210, 240, 270, 300, 330 & 360 min). The graphs were plotted by considering (a-c) time versus growth OD and (g-i) time versus CFU. (d-f) Representative plate images showcasing growth pattern of *Candida* strains during treatment with 3BP at various time interval.

Based on 3BP’s antifungal data, the rate of killing of 3BP at different time intervals was examined by subjecting the fungal strains to a time-killing kinetics experiment. By comparing the viable cell count, growth OD at the given time interval, the graph showcasing the dynamic relationship between the concentration of 3BP and its efficacy over time was plotted (Fig 3). The obtained log_10_ CFU/mL and growth OD of *Candida* spp., exposed to 3BP (MIC & 2XMIC) at various time points are depicted in Fig 3. The initial inoculum of cells treated with the MIC of 3BP did not change significantly (less than 3 log_10_ CFU/mL) up to 1h exposure, and complete reduction was observed with increasing the time of exposure, as shown in Fig 3, indicating that 3BP had fungistatic effects at short time exposure. However, cells treated with 3BP at 2XMIC showed a total decrease in proliferation after 30 min of exposure. The time killing kinetics made it abundantly evident that the concentration and duration of drug exposure are the two key factors that control the transition between fungistatic and fungicidal actions. The results of this experiment are congruent with the findings of a study by Leite et al., [25], which showed that the transition between fungistatic and fungicidal effects of citral depends on its concentration. With a 3BP exposure of 2 h for MIC and 30 min with 2XMIC, complete growth suppression in all *Candida* strains was recorded (Fig 3).

### Elucidation of 3BP’s fungicidal action mechanism

#### Neither the cell wall nor the cell membrane are targets for 3BP’s fungicidal efficacy

Understanding the mechanism of action of antimicrobial agents is critical for developing optimal therapeutic options for treating infections caused by resistant microbes when the first-line antibiotic fails [38]. Most of the conventional antifungal agents, including polyenes as well as the recently approved echinocandin classes, exhibit toxicity towards fungal cells by targeting the cell wall or composition [6]. On this perspective, the mechanistic action of 3BP on the *Candida* cell wall was examined by performing an MIC assay in the presence of sorbitol. Sorbitol is an osmotic protectant that stabilises the fungal cell wall under environmental stress, and its presence in the growth medium is expected to raise the compound’s MIC if it targets the fungal cell wall [26]. This result showed that the MIC value of 3BP in both the presence and absence of sorbitol was identical, clearly signifying that 3BP does not target the fungal cell membrane but rather works on another cell target.

Further, it was hypothesized that conventional antifungal compounds exert killing by targeting ergosterol, a key component of fungal cell membranes that is essential for fluidity and permeability [40]. The clinically approved polyenes including amphotericin-B, nystatin, natamycin, rimocidin, filipin and candicin, all demonstrated cytotoxicity against fungal cells with a strong affinity for fungal ergosterol [41]. Hence, to prove this speculation, the capability of 3BP to bind to ergosterol was investigated by introducing exogenous ergosterol into the medium containing 3BP and thereafter monitoring changes in MIC. Exogenous ergosterol increases the MIC of an antifungal agent that binds to ergosterol by inhibiting molecules from binding to it in cell membranes [42]. The MIC of 3BP did not vary significantly in the presence or absence of ergosterol, signifying that the mode of action of 3BP does not involve binding with ergosterol. The results of the sorbitol and ergosterol assays revealed that neither the fungal cell membrane nor the components of the cell wall are involved in the antifungal mechanism of action of 3BP.

#### 3BP (at MIC) accumulates ROS in *Candida* cells

Many antimicrobial drugs have been well demonstrated to destroy harmful microorganisms by boosting endogenous ROS generation. Along with their unique method of action, every antifungal drug is known to eventually cause the formation of ROS in fungal cells. For instance, miconazole, the azole class antifungal drug known to cause killing by interfering with fungal ergosterol, also causes the production of ROS accumulation in fungal cells [43]. A previous study on 3BP found that it made human colon cancer cells more vulnerable to apoptosis by boosting ROS production [44].

**Fig 4:**
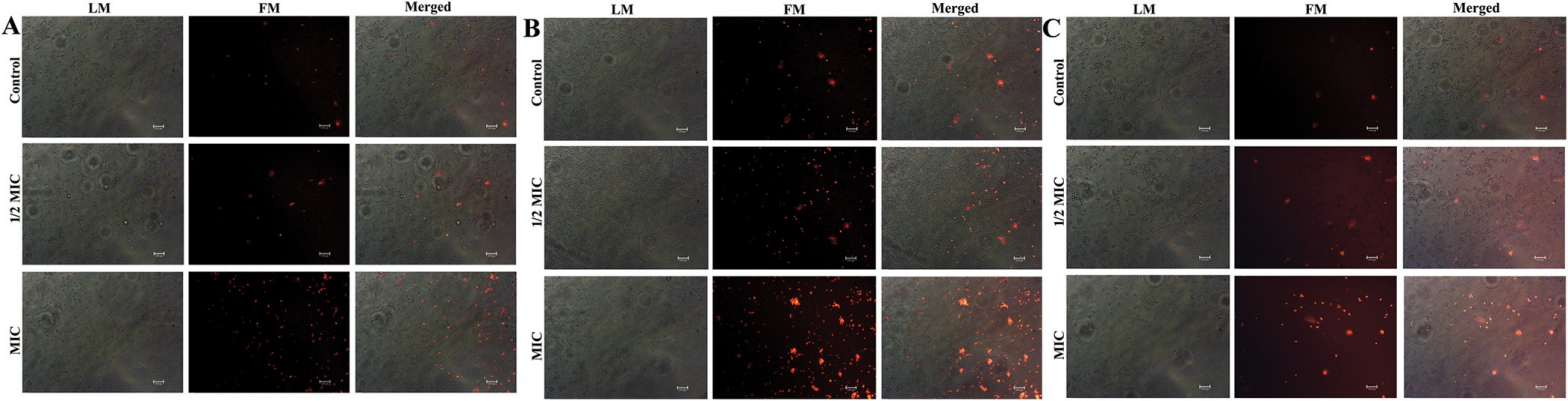
Analyzing cell membrane integrity in all tested *Candida* strains through PI staining (magnification: ×200, scale bar 50 μm). a- *C*. *albicans*; b- *C*. *tropicalis*; c. *C*. *glabrata*.

To ascertain the potential of 3BP exposure to induce ROS-dependent apoptosis, the accumulation of ROS in the *Candida* strain was quantified utilizing a fluorescent probe (DCFH-DA). The fluorescence intensity of cells expressed with 3BP increased significantly as compared to control cells (Fig 4). Additionally, it was noted that the fluorescence intensity increased along with the increasing concentration of 3BP, signifying the concentration dependent ROS accumulation in all tested *Candida* species. Previous report has demonstrated that subjecting *C*. *neoformans* to 3BP increased the production of ROS and consequently caused cell death, which is in consistency with the data of current study [45]. In agreement with the results of the present study, investigations by Seyedjavadi et al (2020) [46], Kobayashi et al (2002) [47] documented ROS accumulation as the key cellular event in the antagonistic activity of antifungal compound. Thus, it is envisaged that 3BP-mediated ROS accumulation could plausibly induce apoptosis in *Candida* species.

#### 3BP achieves fungicidal action by mediating the cell membrane integrity loss of *Candida*

**Fig 5:**
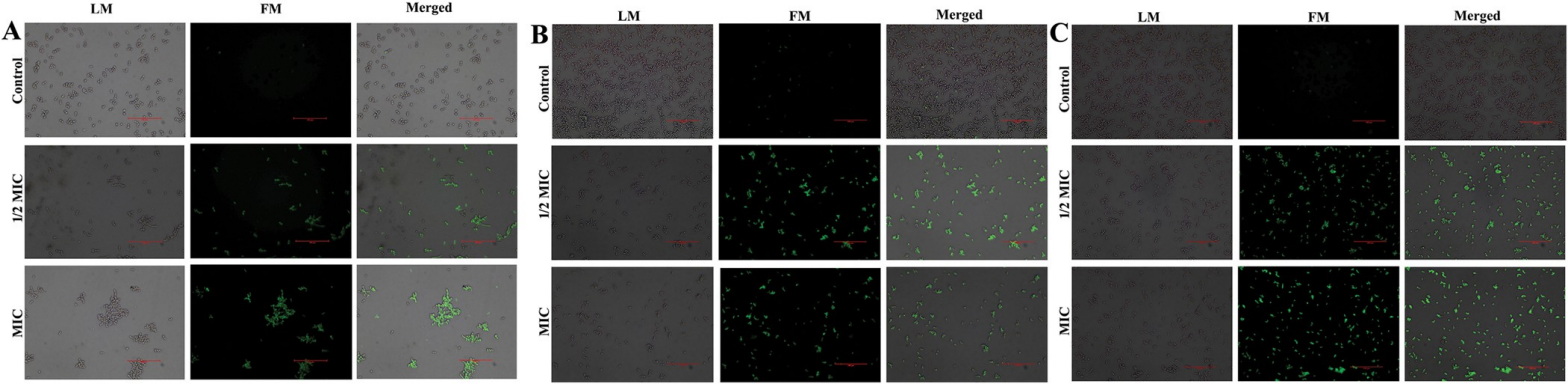
Assessment of intracellular ROS accumulation in *Candida* cells upon manifestation with 3BP (at 1/2MIC & MIC) using DCFH-DA staining (magnification: ×200, scale bar 50 μm). a- *C*. *albicans*; b- *C*. *tropicalis*; c. *C*. *glabrata*.

To further verify the speculation on the mode of killing activity of 3BP, cell membrane integrity was assessed by employing propidium iodide (PI) staining. PI specifically penetrates and stains DNA in only dead cells or those with damaged cell membranes, and therefore it is used as an indicator of cell membrane integrity. If the drug compromises the cell membrane to cause death, PI can quickly enter the cell and boost fluorescence intensity, which can be seen under a microscope [29]. As shown in Fig 5, cells treated with 3BP at varied doses showed increased fluorescence intensity, indicating that 3BP significantly damaged cell membrane integrity in all tested *Candida* strains.

With the increased PI intensity, it could also be inferred that 3BP treatment renders the externalization of phosphatidylserine (PS), which is an early marker of apoptosis in fungi. The PS of normal/healthy cells is internalized in the inner leaflet; however, cells undergoing apoptosis and necrosis readily expose the PS on the outside leaflet for PI binding [28]. As a result, the data of the current study indicate the possible apoptotic mode of action of 3BP on *Candida* cell death. The findings of the present study are in corroboration with a study on 3BP by Sun et al., [18], wherein they demonstrated a similar mode of action of 3BP against colon cancer cells (SW480 and HT29 cells) through Annexin/PI staining.

#### DAPI staining unveiled nuclear condensation upon 3BP treatment

**Fig 6:**
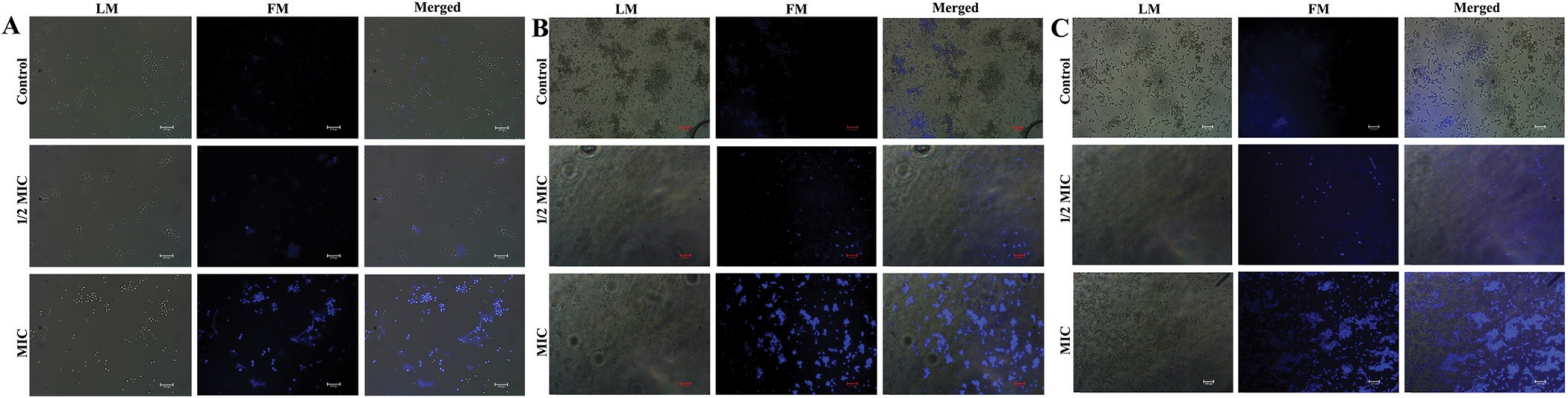
Investigation of nuclear membrane condensation through DAPI staining (magnification: ×200, scale bar 50 μm). a- *C*. *albicans*; b- *C*. *tropicalis*; c. *C*. *glabrata*.

The morphological hallmarks of apoptosis include cell shrinkage, nuclear fragmentation and condensation [29]. Hence, to further gain insights into the apoptotic mode of action of 3BP on *Candida* cells, DAPI staining was performed. A fluorescent interaction dye, DAPI, closely adheres to adenine-thymine-rich regions of DNA, allowing its fluorescence to be detected. The intensity of DAPI increases as a result of drug exposure, compacting the chromatin in nuclei and highlighting abnormally shaped nuclei with a deep blue fluorescence. A fluorescence microscopic study showed that cells that had been treated with 3BP had enhanced deep blue colour intensity, but controls showed a lack of fluorescence intensity, indicating the possibility of nuclear condensation after 3BP treatment (Fig 6).

Overall, the experimental results of microscopic analysis clearly demonstrated the occurrence of PS externalization, nuclear condensation, and ROS accumulation in *Candida* cells upon treatment with 3BP, suggesting that the mechanism underlying the fungicidal action of 3BP could plausibly be ROS-dependent cell death. Numerous investigations on 3BP cytotoxicity demonstrate its apoptotic mode of cell death in different cancer cells, which is consistent with the data currently available. A study by Chen et al., [44] demonstrated tumor necrosis factor-related apoptosis-inducing ligand (TRAIL) linked apoptosis in human breast cancer cells. In addition, yet another study by Nikravesh et al., [48] reported that the 3BP treatment significantly induced apoptosis, activation of caspase 3 activity, depolarization of mitochondrial membrane potential and ROS production in colorectal cancer cell line.

#### 3BP (at MIC) significantly eradicates *Candida’s* mature biofilm

*Candida* species serve as imperative pathogens with the inherent potency to form recalcitrant biofilms those are resistant to conventionally available antifungal drugs [49]. Unlike planktonic cells, sessile biofilm cells vary greatly within the genus *Candida*, providing advantages for their survival through a diversified mechanism of pathogenesis. For instance, the biofilms of *C*. *albicans* and *C*. *tropicalis* are comprised of yeast, pseudohyphae and hyphae, while only yeast cells in multilayer attributes are found in the *C*. *glabrata* biofilm [50]. Due to such variances in biofilm composition, *Candida* species pose a significant challenge in developing new methods for the eradication of their respective biofilm.

**Fig 7:**
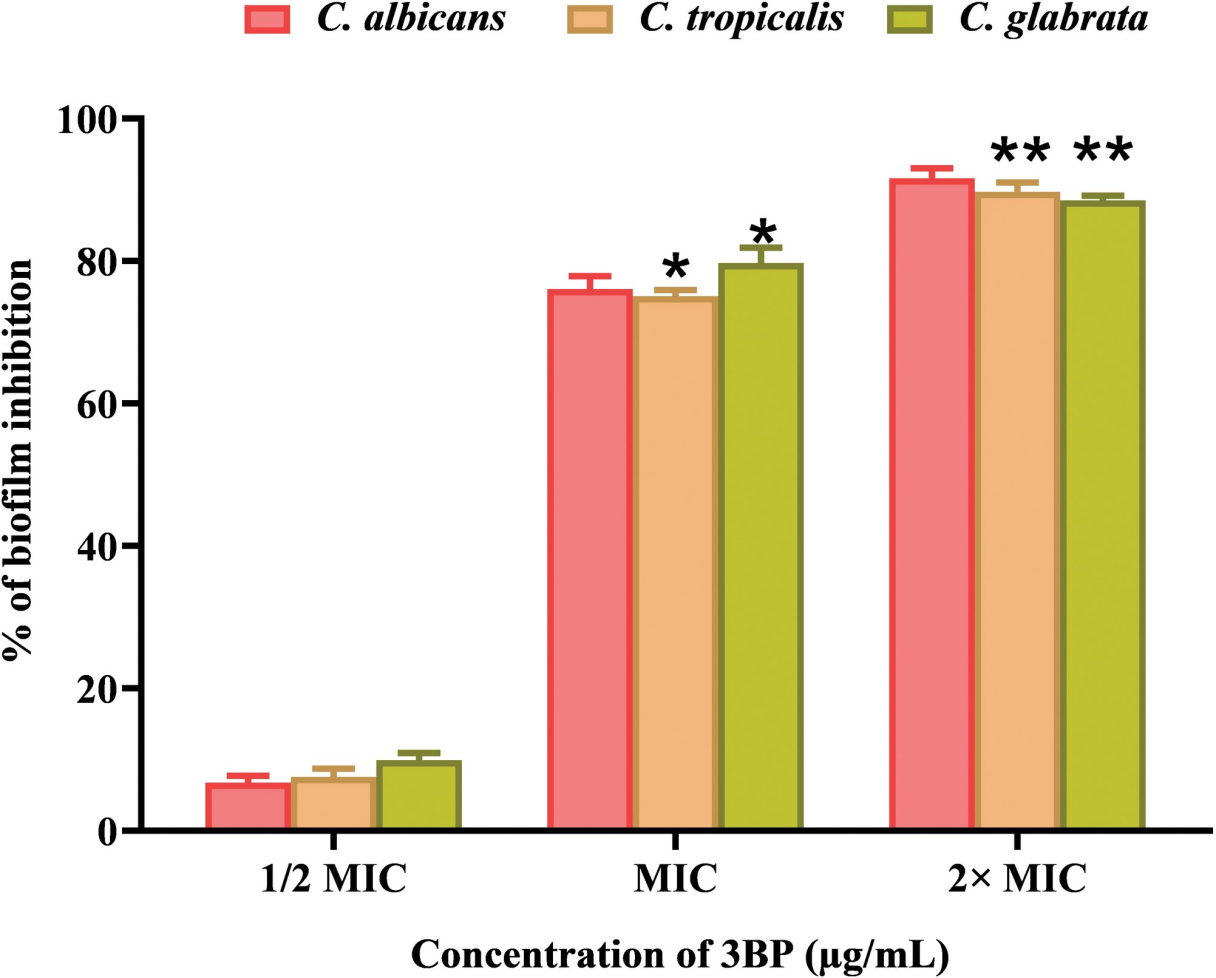
The influence of 3BP on mature biofilm production of *Candida* species using crystal violet staining method. The “* “and “**” symbols represents the statistical significance of p < 0.05 and p < 0.01, respectively.

Besides the immature biofilm, the mature biofilm of *Candida* species are heterogeneous in terms of extracellular components dispersion, making them incredibly robust to the action of conventional antimicrobial agents [51]. For this purpose, the eradication efficacy of 3BP against *Candida* species mature biofilm was assessed using the crystal violet staining method. The spectrometric data showed that 3BP’s at MIC and 2XMIC have enough potency to effectively destroy the pre-formed biofilm to varying degrees—more than 85 and 90%, respectively (Fig 7). However, sub-MICs of 3BP (1/2 MIC) have failed to eradicate mature biofilms of *Candida* species. As discussed before, biofilm production by antimicrobial-resistant pathogens poses significant challenges in disease management. In this regard, the current investigation validated the potential use of 3BP in hospital settings to stop persistent colonisation of fungal infections by revealing a promising fungicidal effect as well as biofilm eradication efficacy. Even though the anticancer and antimicrobial effectiveness of 3BP have been documented through several studies worldwide, the current study is the first of its kind to unveil the biofilm eradication ability of 3BP against an important invasive fungal pathogens, *Candida* species.

#### Non-toxic nature of 3BP in both *in vitro* and *in vivo* conditions

An ideal bioactive compound with clinical application must not be harmful in nature. Therefore, the cytotoxicity of 3BP was investigated using both *in vitro* (human red blood cells) and *in vivo* (*G*. *mellonella*) models.

**Fig 8:**
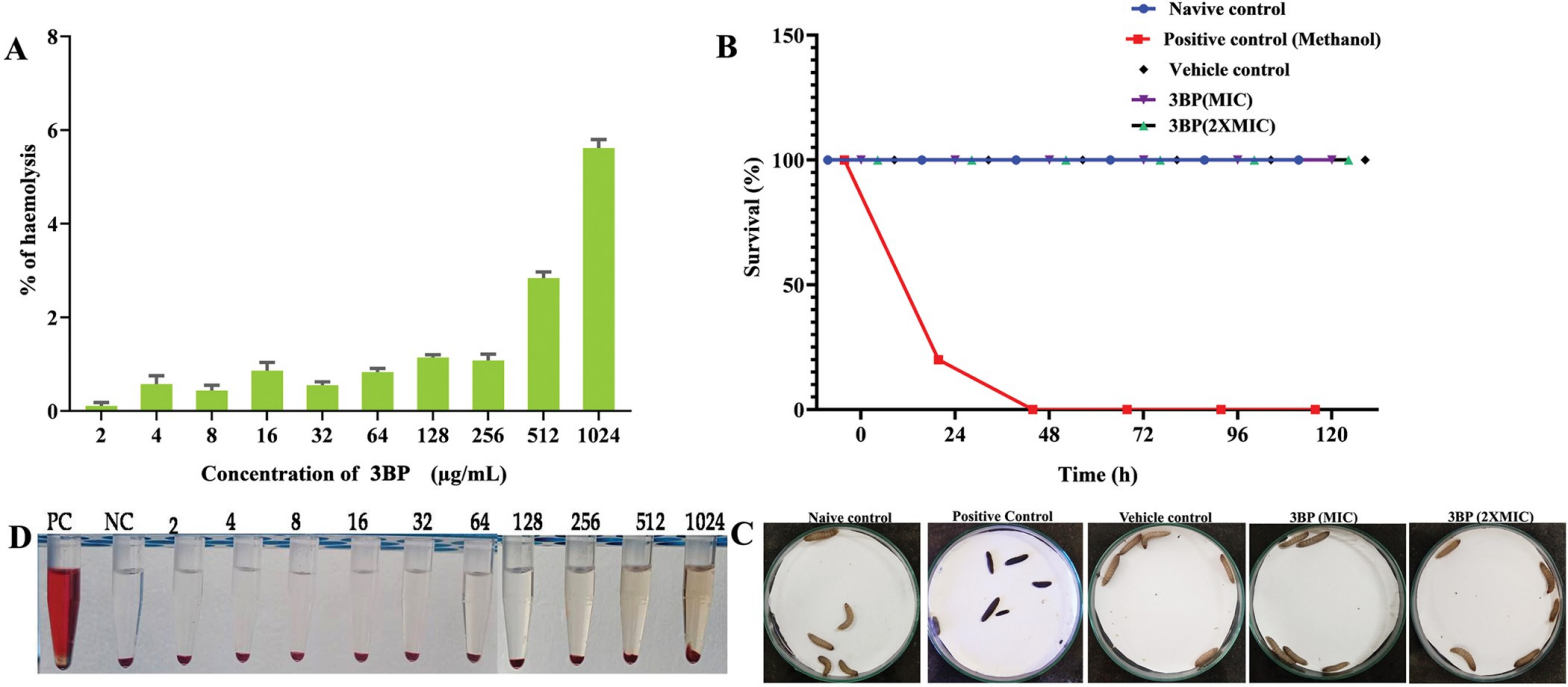
Assessment of 3BP cytotoxicity (a) Impact of 3BP on human erythrocyte cells. No haemolytic activity was observed. The graph was plotted with absorbance by using SDS (10%) as positive control, it seems to completely lysed the total erthryocytes. (b) Representative image showcasing the non-haemolytic activity of 3BP. (c) The survival graph of *G*. *mellonella* manifested with 3BP (at MIC & 2XMIC) for 5 days. (d) The representative image demonstrating the survival of *G*. *mellonella* at the end of the experiment (at 5^th^ day).

In the erythrocyte lysis assay, the wide range of 3BP’s concentrations was subjected to toxicity analysis. The cytotoxicity exhibited at different concentrations of 3BP is shown in Fig 8. The results showed that 3BP did not impose erythrolysis up to a concentration of 512 μg/mL, as clear PBS was seen due to the settling of an intact erythrocyte. Even though 3BP at 1024 μg/mL showed hemolysis, it only represented a very small fraction (8%) compared to the positive control. The presence of a red colour in the positive control (SDS 10%) tube indicated that all red blood cells had been thoroughly lysed. According to a research by Sadowska-Bartosz et al. [52], 3BP has an impact on human erythrocyte cells; however at doses up to 0.75 mM, there are no negative effects on the integrity of the erythrocyte membrane.

In addition, the *in vivo* toxicity of 3BP at MIC and 2XMIC was examined by employing a simple eukaryotic model organism, *G*. *mellonella*. Despite its other advantages, the non-ethical implications of *G*. *mellonella* make it an ideal model for toxicological investigation. The toxicity of 3BP against *G*. *mellonella* was studied using a survival assay. The survivability of larvae at different concentrations of 3BP over 5 days is illustrated in Fig 8. Larvae injected with 3BP at both MIC and 2XMIC did not show any toxicity, as there was no change in the survivability of incubated larvae, demonstrating the non-toxic nature of 3BP (Fig 8). The larvae administered with vehicle control (ethanol) showed no reduction in survival. Interestingly, the larvae exposed to positive control completely hampered survival (100% mortality). Taken together, these findings demonstrated that 3BP is safe for human use, as it had no negative effects on human erythrocytes and *in vivo* model *G*. *mellonella*. Notably, the previous study by Geschwind et al., (2002) [17] revealed the non-toxic nature of 3BP (5mM) on rabbits; they also found that there is no apparent harm to the organs of 3BP implanted rabbits. However, a study by Chang et al., (2007) [53] demonstrated that treatment with 3BP at 25 mM causes hepatotoxicity (including hemorrhagic pyloric or duodenal necrosis) in a rat model. These finding evidently signifies that the cytotoxicity of 3BP is in turn associated with its concentration (dose dependent toxicity).

#### 3BP based vaginal formulation retained its physiological characteristics even after 30 days

The journey of a novel drug from the laboratory to the clinic is rarely simple. Even though multiple studies have documented the phenomenal inhibiting activity of new drugs against drug resistant microorganisms, the clinical application of these identified drugs remains unknown. This is due to the fact that the majority of research has solely concentrated on testing new drugs *in vitro*, rather than on their clinical application. Thus, to ensure the therapeutic usefulness of the discovered 3BP molecules against VVC, the current study was expanded to design a formulation for 3BP-loaded vaginal cream.

Although VVC is more readily with oral agents than topical antifungals, issues pertaining to the oral route, *viz*., systemic toxicity and drug interactions, restrict its usage, which leads to RVVC [53, 54]. The vaginal route of drug administration is conventionally believed to be an effective delivery approach. Therefore, the current study focused on developing an antifungal vaginal cream incorporated with the identified anti-candidal agent 3BP and testing its efficacy in an *in vitro* as well as *in vivo* mice model against *C*. *albicans*.

The oil/water emulsion method was employed to develop the vaginal cream formulation. In this study, the formulation containing 2% miconazole was used as a positive control. Initially the formulated vaginal cream was checked for its stability. The macroscopic observation of the current study revealed that the formulated cream retained its colour, odour and texture throughout the 30 days of analysis. Moreover, the pH of the formulated cream did not alter much; it remained within the acceptable range of vaginal pH (3–4.5). Since the innate immune system is significantly influenced by the vaginal pH, the unchanged pH of the formulated cream ensures its favourable behavior for vaginal application. The cream formulations’ spreadability was measured to be between 1.5 and 1.8 cm in diameter and did not change over time, indicating that the cream’s consistency and moisture content also remained unaltered.

The efficacy of a formulated vaginal cream containing 3BP in inhibiting *Candida* growth was assessed by a well-diffusion assay. In this experiment, the only organism employed to evaluate the efficacy of 3BP cream was *C*. *albicans*. As the determined MIC of drugs may fluctuate depending on the conditions, the various concentrations of 3BP in the vaginal cream (0.25, 0.5 and 1%) were checked for their antifungal efficacy against *C*. *albicans*. The results showed that 1% 3BP cream had significantly greater fungicidal efficiency than 0.25 and 0.5% of cream, respectively. Through a broth microdilution test, the MIC of 3BP was found to be 256 μg/mL (0.025%). The 3BP active concentration in vaginal cream was found to be 1%, proving that the drug’s estimated MIC changed depending on the nature of the medium used. Therefore, it is highly recommended to ascertain a drug’s MIC in its therapeutic form.

**Fig 9:**
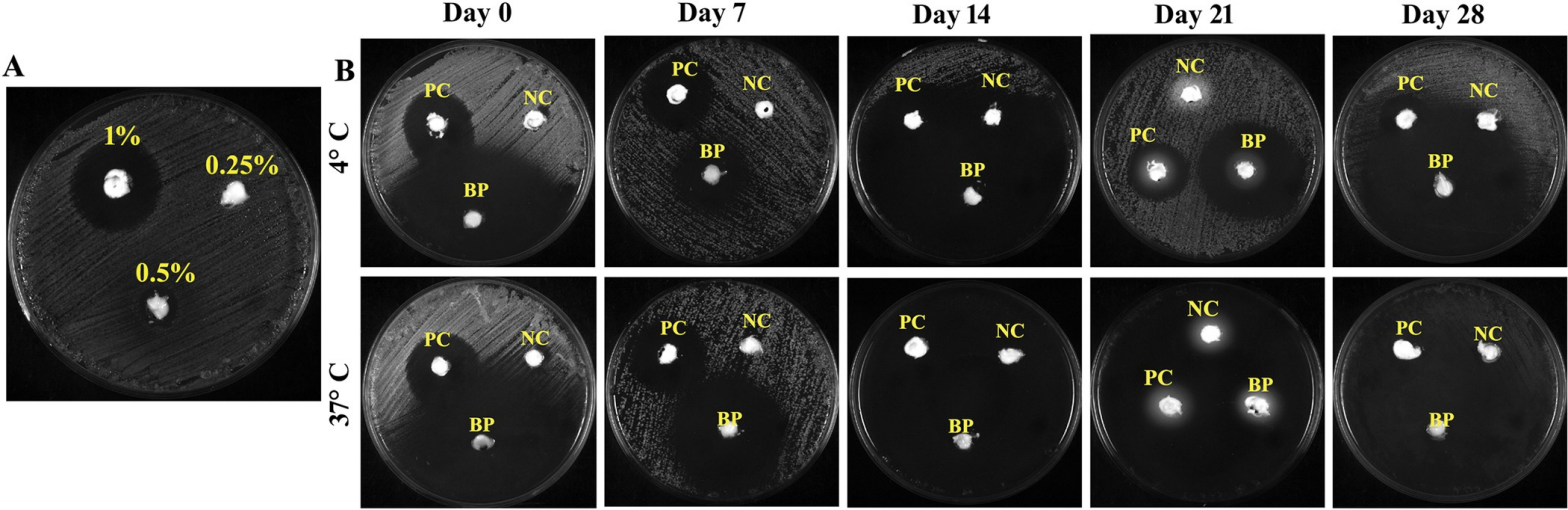
*In vitro* efficacy of 3BP loaded vaginal cream formulation. Cream containing 2% miconazole served as positive control. (a) Optimization of 3BP various concentrations (0.25, 0.5 & 1%) against *C*. *albicans* growth (b) Antifungal efficacy of formulated vaginal creams against *C*. *albicans* under *in vitro* conditions. PC, NC, BP represented in the plates denotes the positive control (miconazole), negative control (cream formulation devoid of active agents), 3BP containing creams, respectively.

The stability of the formulated cream in displaying fungicidal efficacy was determined over a 30-day of period with a 7-day time interval at two different temperatures. The obtained result showed that 3BP retained its efficacy in inhibiting *C*. *albicans* for upto 30 days (Fig 9). In terms of temperature, the 3BP cream held at 37°C appears to have greater antifungal efficacy than those stored at 4°C. However, there was no discernible variation in the fungicidal activity of the vaginal cream positive control that included miconazole, indicating that the 3BP has a preferred temperature of 37°C for its promising antifungal effectiveness. Overall, the proven physiochemical and fungicidal stability of the formulated vaginal cream under different ambient conditions signifies that the optimized vaginal cream formulation could be suitable for the vaginal drug delivery system.

#### Treatment with 3BP vaginal cream reduces fungal burden and protects the membrane integrity of epithelial cells

The *in vivo* antifungal efficacy of a vaginal cream containing 1% of 3BP was assessed in an immunosuppressed and estrogen-dependent mice model. This model demonstrated the pseudo-estrus state, which is required for the establishment of *C*. *albicans* infection by suppressing the innate immune response.

**Fig 10:**
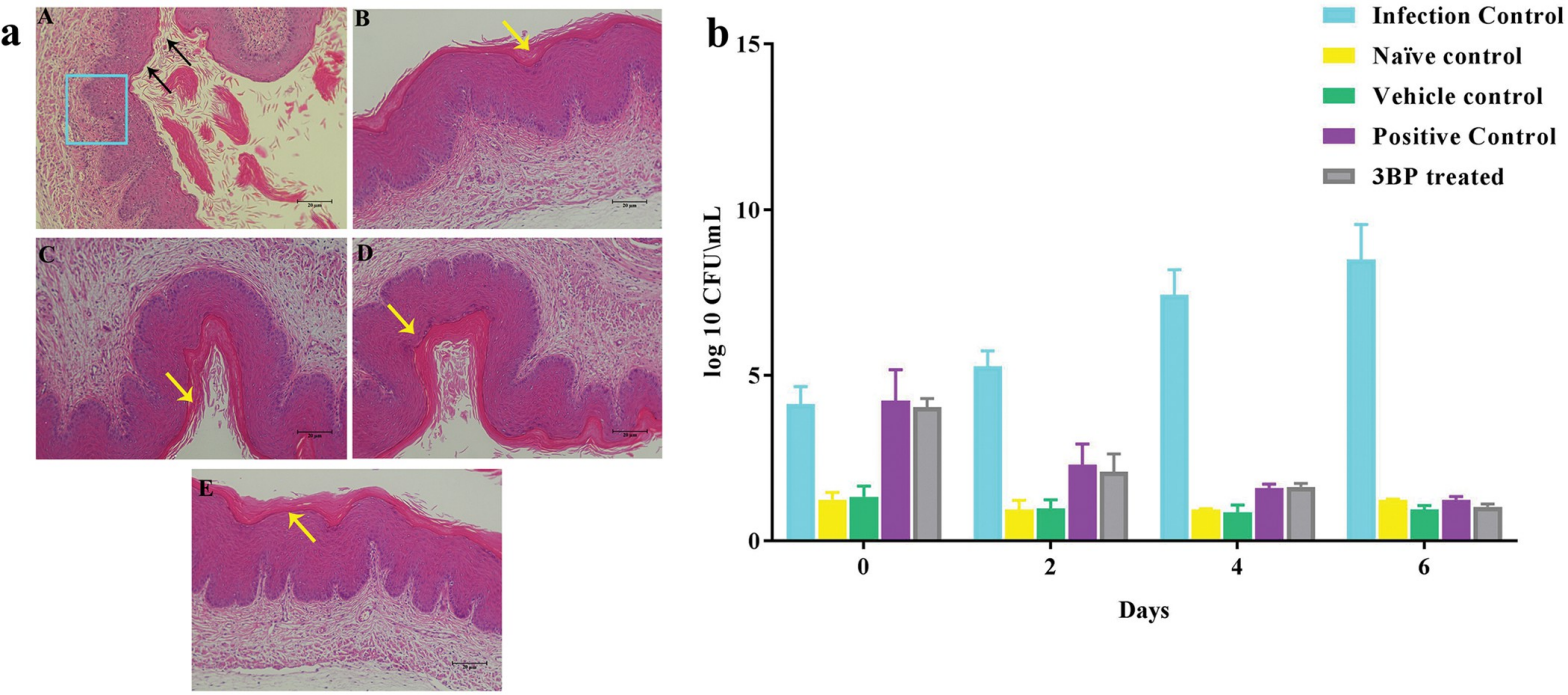
**(a)** *In vivo* efficacy checking of 3BP cream in VVC mice model. (A) Micrograph image demonstrates the appearance of vaginal tissue analyzed after hematoxylin-eosin staining. (A) infected control group (infection control), (B) non- infected animals (naive control), and (C) non- infected and treated with 3BP cream (toxicity control), (D) infected and treated with miconazole cream (vehicle control), infected and treated with 3BP cream (treatment control). Black colored arrows indicate the loss of membrane integrity upon *C*. *albicans* infection. Whereas red colored arrows denote intact vaginal membrane of mice vagina treated with both miconazole and 3BP containing cream. Impaired vaginal epithelium is designated in blue colored box in infected control group. (b) Fungal burden evaluated through CFU counting.

The determined fungal burden in both treated and untreated mice was shown as CFU/mL in Fig 10. The data showed that 3BP vaginal cream effectively reduces the fungal burden in a similar fashion to miconazole cream. As seen in Fig 10, the vaginal load in the infected control group appears to be rising after 2 days of *C*. *albicans* culture establishment. However, the established *C*. *albicans* fungal load was gradually reduced to a level of two log10 in the 3BP and miconazole cream treated groups. Also, there was no complete reduction in the load of fungal cells observed after 6 days of treatment with 3BP and miconazole containing cream.

In order to investigate the activity of 3BP cream in protecting fungal vaginal epithelium cells from the infection induced by *C*. *albicans*, histopathological analysis was done. The micrograph taken from a histopathological sample of 3BP treated and uninfected animals showed intact/undamaged cells with a thick layer of vaginal epithelial membrane. However, the *C*. *albicans* infected samples exposed a loss of epithelium membrane integrity with highly collapsed epithelial membranes (Fig 10). In addition, there was no damage/inflammation detected in the vaginal epithelium of the vehicle control group (animals exposed to 3BP cream alone), signifying the non- toxic nature of 3BP containing cream over fungal vaginal cells. Overall, the 3BP cream was found to be efficient at reducing fungal load and protecting vaginal membrane integrity from *C*. *albicans* infection. Even though the treatment did not completely eliminate the fungal burden, extending the treatment period beyond 6 days will resolve this condition.

## Conclusion

Data of the present study delineates the promising anticandidal potential of 3BP, a well-established anticancer medication, against three clinically significant *Candida* species. Together, our findings provide mechanistic insights into the underlying anticandidal action of 3BP. In *Candida* cells, 3BP induced membrane integrity loss, nuclear condensation and ROS amplification. Additionally, this study displays the *in vivo* anti-infective efficacy of 3BP as a vaginal cream in a mice model of VVC. Overall, the present research clearly demonstrates the feasibility of repurposing anticancer drugs into effective antifungal agents, offering a convincing therapeutic alternative against *Candida* infections, particularly VVC.

## Supporting information

S1 Data (XLS)

## References

[1] Senthilganesh J., Ravichandran S., Durairajan R., BalaSubramaniyan S., Krishnasamy L., Veerappan A., et al. (2021). Metal sulfide nanoparticles based phytolectin scaffolds inhibit vulvovaginal candidiasis causing *Candida albicans*. *Journal of Cluster Science*, 1–12.

[2] Kord Z., Fata A., & Zarrinfar H. (2017). Molecular Identification of *Candida* species isolated from patients with vulvovaginitis for the first time in Mashhad. The *Iranian Journal of Obstetrics*, *Gynecology and Infertility*, 20(4), 50–57.

[3] Alizadeh M., Kolecka A., Boekhout T., Zarrinfar H., Nahzag M. A. G., Badiee P., et al. (2017). Identification of *Candida* species isolated from vulvovaginitis using matrix assisted laser desorption ionization-time of flight mass spectrometry. *Current medical mycology*, 3(4), 21. 10.29252/cmm.3.4.21 PMC5917097 29707675

[4] Achkar J. M., & Fries B. C. (2010). *Candida* infections of the genitourinary tract. *Clinical microbiology reviews*, 23(2), 253–273. 20375352 10.1128/CMR.00076-09 PMC2863365

[5] Minooeianhaghighi M. H., Sehatpour M., Zarrinfar H., & Sen T. (2020). Recurrent vulvovaginal candidiasis: the causative agents, clinical signs and susceptibility to fluconazole in Gonabad city, northeast Iran. *Current Women’s Health Reviews*, 16(1), 46–51.

[6] Chaabane F., Graf A., Jequier L., & Coste A. T. (2019). Review on antifungal resistance mechanisms in the emerging pathogen *Candida auris*. *Frontiers in microbiology*, 10, 2788. 31849919 10.3389/fmicb.2019.02788 PMC6896226

[7] Roemer T., & Krysan D. J. (2014). Antifungal drug development: challenges, unmet clinical needs, and new approaches. *Cold Spring Harbor perspectives in medicine*, 4(5), a019703. doi: 10.1101/cshperspect.a019703 24789878 PMC3996373

[8] Greer N. D. (2003, April). Voriconazole: the newest triazole antifungal agent. In Baylor University Medical Center Proceedings (Vol. 16, No. 2, pp. 241–248). *Taylor & Francis*. 10.1080/08998280.2003.11927910 PMC1201014 16278744

[9] Zarnowski R., Sanchez H., Covelli A.S., Dominguez E., Jaromin A., Berhardt J., et al., 2018. *Candida albicans* biofilm–induced vesicles confer drug resistance through matrix biogenesis. *PLoS biology*, 16(10), p.e2006872. 30296253 10.1371/journal.pbio.2006872 PMC6209495

[10] Sanguinetti M., Posteraro B. and Lass‐Flörl C., 2015. Antifungal drug resistance among *Candida* species: mechanisms and clinical impact. *Mycoses*, 58, pp.2–13. 10.1111/myc.12330 26033251

[11] Nett J.E., Cain M.T., Crawford K. and Andes D.R., 2011. Optimizing a *Candida* biofilm microtiter plate model for measurement of antifungal susceptibility by tetrazolium salt assay. *Journal of clinical microbiology*, 49(4), pp.1426–1433. 21227984 10.1128/JCM.02273-10 PMC3122839

[12] Moghadam S., Azari B., Rashidi R., Bafghi M. H., Rakhshandeh H., Selman S. M., et al. (2023). Antifungal activity of three different varieties of Capsicum annuum against clinical isolates of *Candida* species. *Tropical Diseases*, *Travel Medicine and Vaccines*, 9(1), 9. 37468970 10.1186/s40794-023-00194-w PMC10357713

[13] Nile C., Falleni M., Cirasola D., Alghamdi A., Anderson O.F., Delaney C., et al., 2019. Repurposing pilocarpine hydrochloride for treatment of *Candida albicans* infections. Msphere, 4(1). 10.1128/mSphere.00689-18 PMC6344604 30674648

[14] Visca P., Pisa F., & Imperi F. (2019). The antimetabolite 3-bromopyruvate selectively inhibits *Staphylococcus aureus*. *International journal of antimicrobial agents*, 53(4), 449–455. 30472291 10.1016/j.ijantimicag.2018.11.008

[15] Rangel-Vega A., Bernstein L. R., Mandujano-Tinoco E. A., García-Contreras S. J., & García-Contreras R. (2015). Drug repurposing as an alternative for the treatment of recalcitrant bacterial infections. *Frontiers in microbiology*, 6, 282. doi: 10.3389/fmicb.2015.00282 25914685 PMC4391038

[16] Rampioni G., Visca P., Leoni L., & Imperi F. (2017). Drug repurposing for antivirulence therapy against opportunistic bacterial pathogens. *Emerging Topics in Life Sciences*, 1(1), 13–22. doi: 10.1042/ETLS20160018 33525812

[17] Geschwind J. F. H., Ko Y. H., Torbenson M. S., Magee C., & Pedersen P. L. (2002). Novel therapy for liver cancer: direct intraarterial injection of a potent inhibitor of ATP production. *Cancer research*, 62(14), 3909–3913. 12124317

[18] Sun Y., Liu Z., Zou X., Lan Y., Sun X., Wang X., et al. (2015). Mechanisms underlying 3-bromopyruvate-induced cell death in colon cancer. *Journal of bioenergetics and biomembranes*, 47(4), 319–329. doi: 10.1007/s10863-015-9612-1 26054380 PMC4546694

[19] Feldwisch-Drentrup H. (2016). Candidate cancer drug suspected after death of three patients at an alternative medicine clinic. *Science*, 12.

[20] Azevedo-Silva J., Queirós O., Baltazar F., Ułaszewski S., Goffeau A., Ko Y. H., et al. (2016). The anticancer agent 3-bromopyruvate: a simple but powerful molecule taken from the lab to the bedside. *Journal of bioenergetics and biomembranes*, 48(4), 349–362. doi: 10.1007/s10863-016-9670-z 27457582

[21] Geschwind J. F., & Vali M. (2015). U.S. Patent Application No. 14/836,649.

[22] Gowrishankar S. and Pandian S.K. Modulation of *Staphylococcus epidermidis* (RP62A) extracellular polymeric layer by marine cyclic dipeptide-cyclo (l-leucyl-l-prolyl) thwarts biofilm formation. *Biochimica et BiophysicaActa (BBA)-Biomembranes*. 1859, 1254–1262 (2017). 10.1016/j.bbamem.2017.04.009 28414038

[23] Hafidi Z., El Achouri M., Sousa O., F. F., & Perez L. (2022). Antifungal activity of amino-alcohols based cationic surfactants and *in silico*, homology modeling, docking and molecular dynamics studies against lanosterol 14-α-demethylase enzyme. *Journal of Biomolecular Structure and Dynamics*, 40(17), 7762–7778. 33754947 10.1080/07391102.2021.1902396

[24] Öz Y., Özdemir H. G., Gökbolat E., Kiraz N., Ilkit M., & Seyedmousavi S. (2016). Time-kill kinetics and *in vitro* antifungal susceptibility of non-fumigatus *Aspergillus* species isolated from patients with ocular mycoses. *Mycopathologia*, 181(3), 225–233. 26612621 10.1007/s11046-015-9969-z PMC4786614

[25] Leite M. C. A., Bezerra A. P. D. B., Sousa J. P. D., Guerra F. Q. S., & Lima E. D. O. (2014). Evaluation of antifungal activity and mechanism of action of citral against *Candida albicans*. *Evidence-Based Complementary and Alternative Medicine*, 2014. 10.1155/2014/378280 PMC4163309 25250053

[26] Leite M. C. A., de Brito Bezerra A. P., de Sousa J. P., & de Oliveira Lima E. (2015). Investigating the antifungal activity and mechanism (s) of geraniol against *Candida albicans* strains. *Medical mycology*, 53(3), 275–284. 25480017 10.1093/mmy/myu078

[27] Jia C., Yu Q., Xu N., Zhang B., Dong Y., Ding X., et al. (2014). Role of TFP1 in vacuolar acidification, oxidative stress and filamentous development in *Candida albicans*. *Fungal Genetics and Biology*, 71, 58–67. 25220074 10.1016/j.fgb.2014.08.012

[28] Jia C., Zhang J., Yu L., Wang C., Yang Y., Rong X., et al. (2019). Antifungal activity of coumarin against *Candida albicans* is related to apoptosis. *Frontiers in Cellular and Infection Microbiology*, 8, 445. 30662877 10.3389/fcimb.2018.00445 PMC6328497

[29] Hao B., Cheng S., Clancy C. J., & Nguyen M. H. (2013). Caspofungin kills *Candida albicans* by causing both cellular apoptosis and necrosis. *Antimicrobial agents and chemotherapy*, 57(1), 326–332. 23114781 10.1128/AAC.01366-12 PMC3535936

[30] Jothi R., Sangavi R., Kumar P., Pandian S. K., & Gowrishankar S. (2021). Catechol thwarts virulent dimorphism in *Candida albicans* and potentiates the antifungal efficacy of azoles and polyenes. *Scientific Reports*, 11(1), 1–20. doi: 10.1038/s41598-020-79139-8 34702898 PMC8548306

[31] Turecka K., Chylewska A., Kawiak A., & Waleron K. F. (2018). Antifungal activity and mechanism of action of the Co (III) coordination complexes with diamine chelate ligands against reference and clinical strains of *Candida spp*. *Frontiers in microbiology*, 9, 1594. 30072969 10.3389/fmicb.2018.01594 PMC6058090

[32] Ignasiak K., & Maxwell A. (2017). *Galleria mellonella* (greater wax moth) larvae as a model for antibiotic susceptibility testing and acute toxicity trials. *BMC research notes*, 10(1), 1–8. 28851426 10.1186/s13104-017-2757-8 PMC5576310

[33] Aslani A., Asghari G., Darani H. Y., Ghanadian M., & Hosseini F. (2019). Design, formulation, and physicochemical evaluation of vaginal cream containing *Eucalyptus camaldulensis*, *Viola odorata*, and *Mentha piperita* extracts for prevention and treatment of Trichomoniasis. *International Journal of Preventive Medicine*, 10. 10.4103/ijpvm.IJPVM_525_17 PMC6826777 32133097

[34] Chen M. X., Alexander K. S., & B Turecka aki G. (2016). Formulation and evaluation of antibacterial creams and gels containing metal ions for topical application. *Journal of pharmaceutics*, 2016. doi: 10.1155/2016/5754349 27885352 PMC5112321

[35] Elisha I. L., Botha F. S., McGaw L. J., & Eloff J. N. (2017). The antibacterial activity of extracts of nine plant species with good activity against Escherichia coli against five other bacteria and cytotoxicity of extracts. *BMC complementary and alternative medicine*, 17(1), 1–10. 28241818 10.1186/s12906-017-1645-z PMC5329917

[36] Fernandes L. D. S., Amorim Y. M., da Silva E. L., Silva S. C., Santos A. J. A., Peixoto F. N., et al. (2018). Formulation, stability study and preclinical evaluation of a vaginal cream containing curcumin in a rat model of vulvovaginal candidiasis. *Mycoses*, 61(10), 723–730. doi: 10.1111/myc.12762 29517833

[37] Krishnasamy L., Senthilganesh J., Saikumar C., & Nithyanand P. (2021). Biofilm-forming fluconazole-resistant *Candida auris* causing vulvovaginal candidiasis in an immunocompetent patient: a case report. Asian Pacific Journal of Tropical Medicine, 14(2), 94.

[38] Senthilganesh J., Deepak L., Durai R., Narayanan V. H. B., Veerappan A., & Paramasivam N. (2022). Evaluation of lectin nanoscaffold based in-situ gel against vulvovaginal candidiasis causing *Candida* biofilms using a novel ex-vivo model. Journal of Drug Delivery Science and Technology, 74, 103560.

[39] Rudrapal M., Khairnar S. J., & Jadhav A. G. (2020). Drug repurposing (DR): an emerging approach in drug discovery. *Drug Repurposing-Hypothesis*, *Molecular Aspects and Therapeutic Applications*.

[40] Ryder N. S., Frank I., & Dupont M. C. (1986). Ergosterol biosynthesis inhibition by the thiocarbamate antifungal agents tolnaftate and tolciclate. *Antimicrobial agents and chemotherapy*, 29(5), 858–860. doi: 10.1128/AAC.29.5.858 3524433 PMC284167

[41] K Mazu T., A Bricker B., Flores-Rozas H., & Y Ablordeppey S. (2016). The mechanistic targets of antifungal agents: an overview. *Mini reviews in medicinal chemistry*, 16(7), 555–578. doi: 10.2174/1389557516666160118112103 26776224 PMC5215921

[42] Lima I. O., de Medeiros Nóbrega F., de Oliveira W. A., de Oliveira Lima E., Albuquerque Menezes E., Afrânio Cunha F., et al. (2012). Anti-*Candida albicans* effectiveness of citral and investigation of mode of action. *Pharmaceutical Biology*, 50(12), 1536–1541. 23116193 10.3109/13880209.2012.694893

[43] Delattin N., Cammue B. P., & Thevissen K. (2014). Reactive oxygen species-inducing antifungal agents and their activity against fungal biofilms. *Future medicinal chemistry*, 6(1), 77–90. doi: 10.4155/fmc.13.189 24358949

[44] Chen Y., Wei L., Zhang X., Liu X., Chen Y., Zhang S., et al. (2018). 3‑Bromopyruvate sensitizes human breast cancer cells to TRAIL induced apoptosis via the phosphorylated AMPK mediated upregulation of DR5. *Oncology reports*, 40(5), 2435–2444. 30132536 10.3892/or.2018.6644 PMC6151892

[45] Dylag M., Niedzwiecka K., Lis P., Ko Y. H., Pedersen P. L., Goffeau A., et al. (2014, May). 3-bromopyruvate as a potent anticryptococcal drug. *In Mycoses* (Vol. 57, pp. 44–44). 111 RIVER ST, HOBOKEN 07030–5774, NJ USA: WILEY-BLACKWELL.

[46] Seyedjavadi S. S., Khani S., Eslamifar A., Ajdary S., Goudarzi M., Halabian R., et al. (2020). The antifungal peptide MCh-AMP1 derived from *Matricaria chamomilla* inhibits *Candida albicans* growth via inducing ROS generation and altering fungal cell membrane permeability. *Frontiers in microbiology*, 10, 3150. 32038583 10.3389/fmicb.2019.03150 PMC6985553

[47] Kobayashi D., Kondo K., Uehara N., Otokozawa S., Tsuji N., Yagihashi A., et al. (2002). Endogenous reactive oxygen species is an important mediator of miconazole antifungal effect. *Antimicrobial agents and chemotherapy*, 46(10), 3113–3117. doi: 10.1128/AAC.46.10.3113-3117.2002 12234832 PMC128784

[48] Nikravesh H., Khodayar M. J., Behmanesh B., Mahdavinia M., Teimoori A., Alboghobeish S., et al. (2021). The combined effect of dichloroacetate and 3-bromopyruvate on glucose metabolism in colorectal cancer cell line, HT-29; the mitochondrial pathway apoptosis. *BMC cancer*, 21(1), 1–11. 34364387 10.1186/s12885-021-08564-3 PMC8349486

[49] Nourizadeh N., Adabizadeh A., Zarrinfar H., Majidi M., Jafarian A. H., & Najafzadeh M. J. (2019). Fungal biofilms in sinonasal polyposis: the role of fungal agents is notable?. *Journal of Oral and Maxillofacial Surgery*, *Medicine*, *and Pathology*, 31(4), 295–298.

[50] Cavalheiro M., & Teixeira M. C. (2018). *Candida* biofilms: threats, challenges, and promising strategies. *Frontiers in medicine*, 5, 28. 29487851 10.3389/fmed.2018.00028 PMC5816785

[51] Chandra J., Kuhn D. M., Mukherjee P. K., Hoyer L. L., McCormick T., & Ghannoum M. A. (2001). Biofilm formation by the fungal pathogen *Candida albicans*: development, architecture, and drug resistance. *Journal of bacteriology*, 183(18), 5385–5394 11514524 10.1128/JB.183.18.5385-5394.2001 PMC95423

[52] Sadowska‐Bartosz I., & Bartosz G. (2013). Effect of 3‐bromopyruvic acid on human erythrocyte antioxidant defense system. *Cell biology international*, 37(12), 1285–1290. doi: 10.1002/cbin.10160 23881849

[53] Chang J. M., Chung J. W., Jae H. J., Eh H., Son K. R., Lee K. C., et al. (2007). Local toxicity of hepatic arterial infusion of hexokinase II inhibitor, 3-bromopyruvate: *In vivo* investigation in normal rabbit model. *Academic radiology*, 14(1), 85–92. 17178370 10.1016/j.acra.2006.09.059

[54] Senthilganesh J., Kuppusamy S., Durairajan R., Subramaniyan S. B., Veerappan A., & Paramasivam N. (2022). Phytolectin nanoconjugates in combination with standard antifungals curb multi-species biofilms and virulence of vulvovaginal candidiasis (VVC) causing *Candida albicans* and non-albicans *Candida*. *Medical Mycology*, 60(2), myab083. 34958385 10.1093/mmy/myab083

